# Laser Micromachining for Bioelectronics: Past, Present, and Future

**DOI:** 10.1002/smtd.202501560

**Published:** 2025-11-23

**Authors:** J. G. Troughton, C. M. Proctor

**Affiliations:** ^1^ Institute of Biomedical Engineering (IBME) Department of Engineering Science University of Oxford Old Road Campus, Headington Oxford OX3 7DQ UK

**Keywords:** bioelectronics, flexible electronics, laser fabrication, laser micromachining, prototyping

## Abstract

Laser micromachining has emerged over the last decade as a practical alternative to costly, complex photolithography, and to low‐resolution printing, for the manufacture of myriad bioelectronic devices. Here, the introduction and development of laser micromachining for bioelectronics is reviewed, and the direction of travel for this fabrication approach is considered. The authors start with early, semi‐manual work on laser cutting metal and silicon foils for cochlear and retinal implants, discuss the development of true laser‐based lithographic analogues for thin‐film material processing, and look at recent work leveraging the localized photothermal effects of lasers to fundamentally alter material properties. The discussion is framed around the various laser systems employed to achieve specific outcomes to inform the reader's choice of system for their own experimental needs.

## Graphical abstract

Lasers offer a straightforward, flexible, and versatile route to the manufacture of bioelectronic devices, with distinct advantages over alternatives such as photolithography and printing. The key aspects of a laser when considering their use in bioelectronics fabrication are discussed in this review, and the development of laser micromachining within bioelectronics over the last 25 years is showcased.

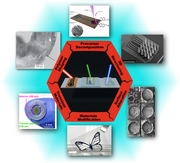

## 1 Introduction

The field of bioelectronics has progressed rapidly over the last 30 years, propelled largely by borrowing from the fabrication techniques and equipment developed for the semiconductor industry.^[^ ^1^ ^]^ This has facilitated the fabrication and miniaturization of myriad clinical and research devices, from cochlear implants^[^ ^2^ ^]^ to closed‐loop diabetes treatments,^[^ ^3^ ^]^ and from brain‐computer interfaces^[^ ^4^ ^]^ to biomimetic computing chips.^[^ ^5^, ^6^ ^]^ However, while the semiconductor industry continues to push for ever‐smaller device sizes in trying to keep up with Moore's Law, much of the bioelectronics world has already reached its goal of achieving device sizes on the scale of animal cells, particularly neuronal cell bodies. Instead, innovations now come predominantly in new materials, new form factors, and new applications. One key technology enabling these innovations is the rise in laser‐based processing.

Laser processing itself has seen a great proliferation over the last 30 years, finding applications in diverse fields from semiconductor manufacturing to home hobby craft. This has been enabled by a rapidly growing commercial field of laser micromachining tools, making laser processing more accessible to both academic and commercial users, as well as lay enthusiasts. The excellent recent review from Pinheiro et al.^[^ ^7^ ^]^ gives a thorough overview of these many applications of laser processing, though only briefly touches on applications in medical and bioelectronic technologies.

**Figure 1:**
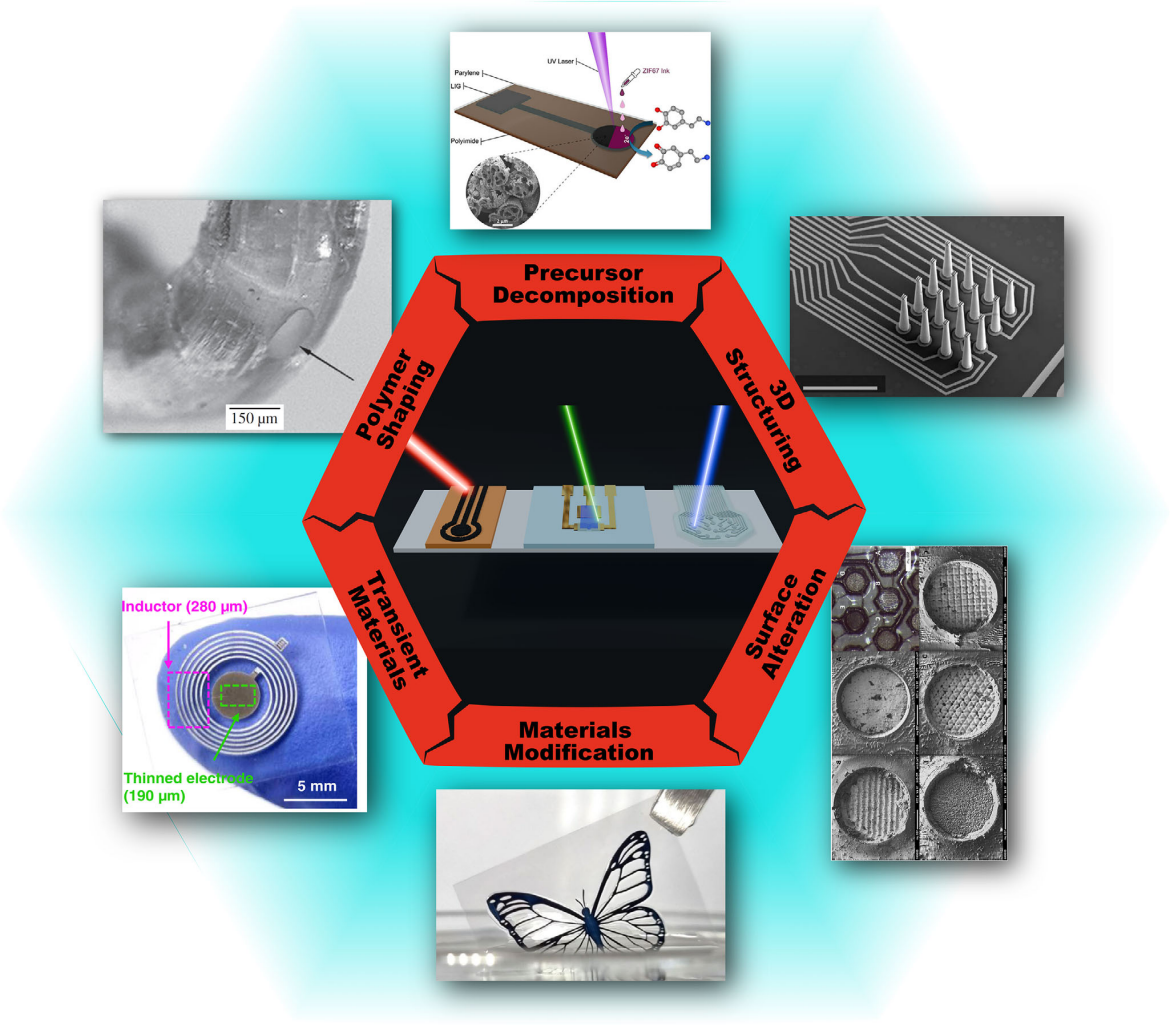
Examples of applications of laser micromachining in bioelectronics covered throughout this review. Clockwise from the top, images show: Laser decomposition of a metal precursor for sensor applications. Reproduced under the terms of the CC‐BY 4.0 license.^[^ ^8^ ^]^ Copyright 2025, De Chiara et al. Opening of 3D printed microelectrode tips for neural interfacing. Reproduced under the terms of the CC‐BY 4.0 license.^[^ ^9^ ^]^ Copyright 2025, Bown et al. Laser structuring of Pt foils for improved neurostimulation, reproduced with permission.^[^ ^10^ ^]^ Copyright 2004, IOP Publishing. Laser‐induced phase separation in PEDOT:PSS for stable neural interfaces, reproduced with permission.^[^ ^11^ ^]^ Copyright 2024, Springer Nature. Laser processing of bioabsorbable materials. Reproduced under the terms of the CC‐BY 4.0 license.^[^ ^12^ ^]^ Copyright 2022, Yang et al. Opening of microfluidic channels in cochlear implants for drug delivery, reproduced with permission.^[^ ^13^ ^]^ Copyright 2013, Taylor & Francis.

In this review, we look at the development of laser machining as a tool for bioelectronics fabrication, covering early works in laser cutting of metal and polymer foils, through microtexturing of electrode surfaces, to the use of laser–matter interaction to alter material properties in unique ways, summarized in **Figure** **1**. The laser parameters key to the applications are discussed, and considerations for the introduction of laser processing to one's workflow highlighted. While many publications feature laser fabrication in their work, a lack of detail in the process description can frustrate efforts to replicate the methods used. In this work, we seek to shine a light on laser processing in bioelectronics, discussing the important details that can make the difference between successful fabrication and a failed run.

## 2 Laser Machining Principles

In order to understand the application of lasers in micromachining, a basic understanding of lasers, laser–matter interaction, and laser machining systems is necessary. Here, a brief description of these follows, though it should be emphasized that this is only an introduction and readers wanting a more thorough treatment of these topics are directed to the review of Pinheiro et al.^[^ ^7^ ^]^ or the many textbooks on laser processing.^[^ ^14^, ^15^, ^16^ ^]^

**Figure 2:**
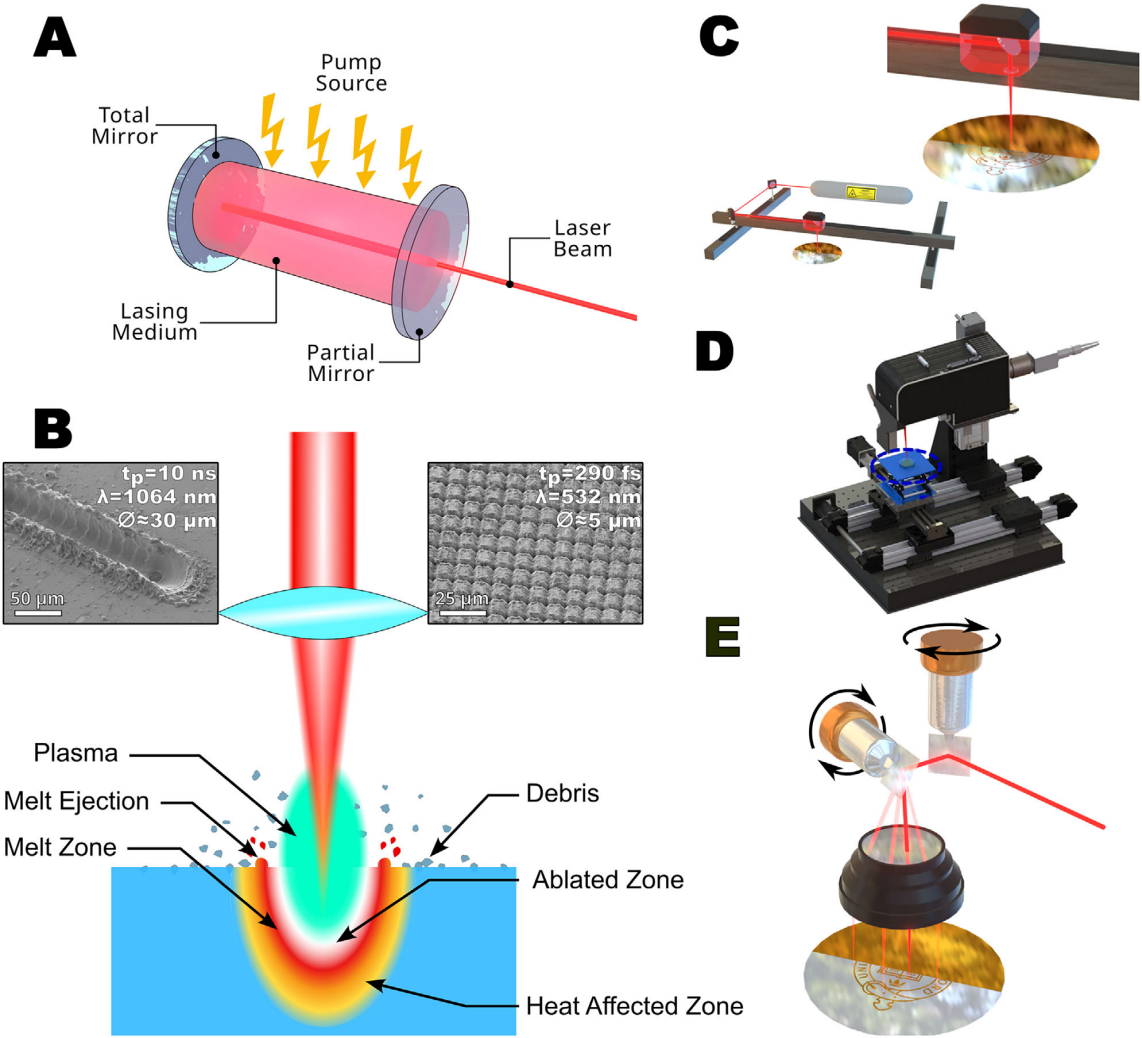
A) Overview of a basic laser source and components. The pump source may be electrical stimulation, a flash lamp, or diode lasers. B) Schematic diagram of thermal laser–material interaction mechanisms. Inset are SEM images of 20 µm thick stainless steel foil processes with a short‐pulse laser (left) and an ultrashort‐pulse laser (right)—the left image shows a melted region and evidence of melt ejection, while the right shows neither. C) Typical laser system based on movement of the laser head, commonly used with CO_2_ laser systems. D) Laser system using bed motion only, commonly used fiber lasers, reproduced with permission.^[^ ^19^ ^]^ Copyright 2022, American Chemical Society. E) Galvanometric laser system with an F‐Theta lens to give a flat‐field view.

Laser light is produced through a cascade process in a lasing medium through repeated excitation and relaxation of the atoms. By pumping energy into the medium, confining the resulting radiation in a mirrored cavity, and controlling the release of the radiation from one end, a coherent, monochromatic, and collimated beam of radiation is produced (**Figure** **2**A). It is these three properties that make laser light unique in optics, and allow its use in device fabrication.

### 2.1 Laser–Matter Interaction

A detailed discussion of the many mechanisms in play during laser micromachining is beyond the scope of this review, with several textbooks dedicated to the subject.^[^ ^17^, ^18^ ^]^ However, a brief discussion of the most dominant effects is important for understanding the process, and the likely impact of laser parameters on the produced devices.

There are two principal effect categories in laser machining: photothermal effects and photochemical effects, with one or both of these occurring for any given material. For both effects, the process starts with the material absorbing photon energy, dependent on the wavelength of the laser light, and both require a minimum, or threshold, laser energy density in order to initiate removal of material.

*Photothermal* effects concern the heating of materials within the interaction volume of the laser beam, and the main features of this are summarized in Figure 2B (note that some, all, or none of the features of Figure 2B may be present depending on the laser and material used). Below the threshold energy density, the material is heated, but no ablation occurs. Depending on the material being processed, there may be some level of material changes, such as disruption of the local structure, but no change in thickness can be measured. As the laser energy density is raised above the threshold, the surface of the material may start to melt, and then vaporize, or it may go directly from the solid to the vapor phase depending on the interaction time of the laser with the material (the pulse length, *t*_p_) and the material properties. Following this, several further processes may occur.

If the material melts and then vaporizes, some of the material is removed simply as a vapor, while additional material may be removed through melt ejection caused by the pressure of the vapor coming from the sample. This mostly occurs with longer pulse lasers (*t*_p_ > 1 ns), but is avoided for ultrashort‐pulse systems. This melt ejection is a particular challenge in laser micromachining as it is a major contributor to unwanted redeposition of material across the sample surface and the buildup of a burr at the edge of the cut (see the inset of Figure 2B for an example of this).

While the creation of a vapor plume itself has little effect on machining, if the laser energy density is high enough this vapor may become ionized to create a plasma. This plasma can have two significant effects depending on the position of the plasma relative to the sample surface. If the laser energy is only slightly above the ionization threshold, the plasma remains coupled to the sample surface. In this case, the plasma can act to enhance the efficiency of material removal, both by increasing the absorption of the incident laser radiation, and by more efficiently promoting alternative ablation mechanisms (e.g., photochemical), though the plasma also reduces the achievable minimum feature size due to its extended size. If the laser energy is even higher, the whole vapor plume may become ionized, and also have sufficient kinetic energy to move away from the sample surface. In this case, almost all of the laser energy is absorbed by the plasma, shielding the workpiece and preventing further material removal. This is known as plasma shielding, and is a particular challenge when trying to laser drill deep holes. As with melting, the use of ultrashort‐pulse lasers can effectively prevent the generation of a plasma, since the pulse length is significantly shorter than the time needed to generate the plasma.

The final photothermal mechanism to consider is explosive ablation. This can occur within a melt region, particularly with shorter pulse lasers, where rapid vaporization causes bubbles to form and explode, ejecting molten material. Alternatively, explosions can be caused by thermally induced stress mismatches (particularly common in multilayer thin‐film systems), where the rapid local heating causes a buildup of stress in one layer, culminating in explosive material ejection. As with melt ejection, explosive ablation often leads to redeposition of ablation debris across the sample surface.

*Photochemical* effects are the other route through which material may be removed from a sample. Here, the energy of incident photons directly breaks chemical bonds within the sample, and is most commonly seen in polymers, inorganic insulators, and semiconductors, particular when using high‐energy photons (UV lasers). After bonds are broken, the newly created species are removed from the sample surface, either through their own high volatility or through additional heat‐mediated removal by the laser. Photochemical bond‐breaking may also introduce a significant level of defects within the bulk of the material which then act as effective dopants, introducing additional excitation levels within the bandgap and increasing the optical absorption. This can then increase rate of both photothermal and photochemical processes, leading to further ablation of the material.

### 2.2 Key Parameters

**Figure 3:**
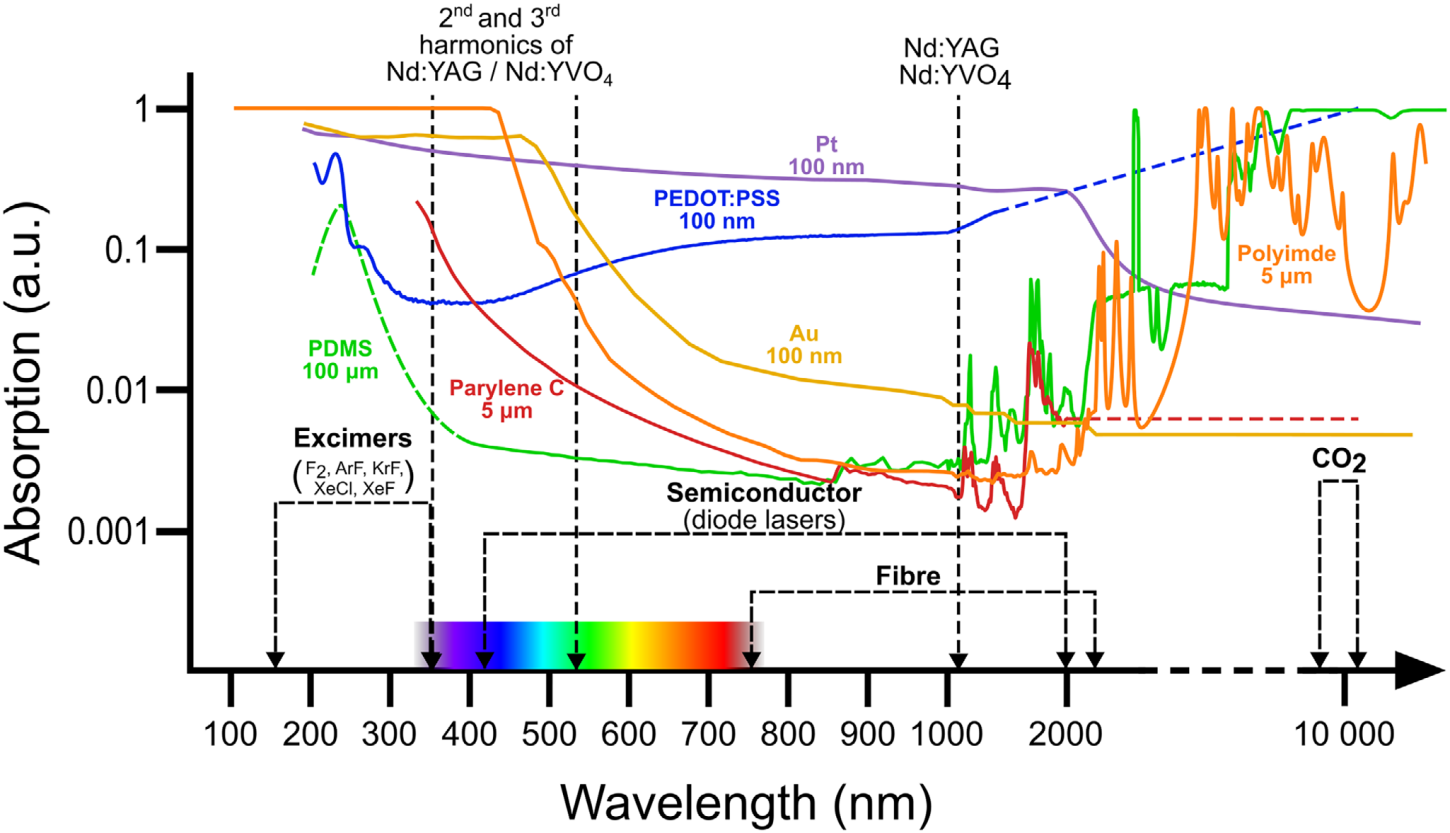
Absorption profiles of several common materials in bioelectronics along with indication of the wavelengths for various laser types. Absorption data are derived from various sources: Au,^[^ ^20^ ^]^ Pt,^[^ ^21^ ^]^ PEDOT:PSS,^[^ ^22^ ^]^ Parylene C,^[^ ^23^ ^]^ PDMS,^[^ ^24^ ^]^ and polyimide.^[^ ^25^, ^26^, ^27^ ^]^ Dashed lines are inferred data from multiple sources and are indicative rather than exact.

**Figure 4:**
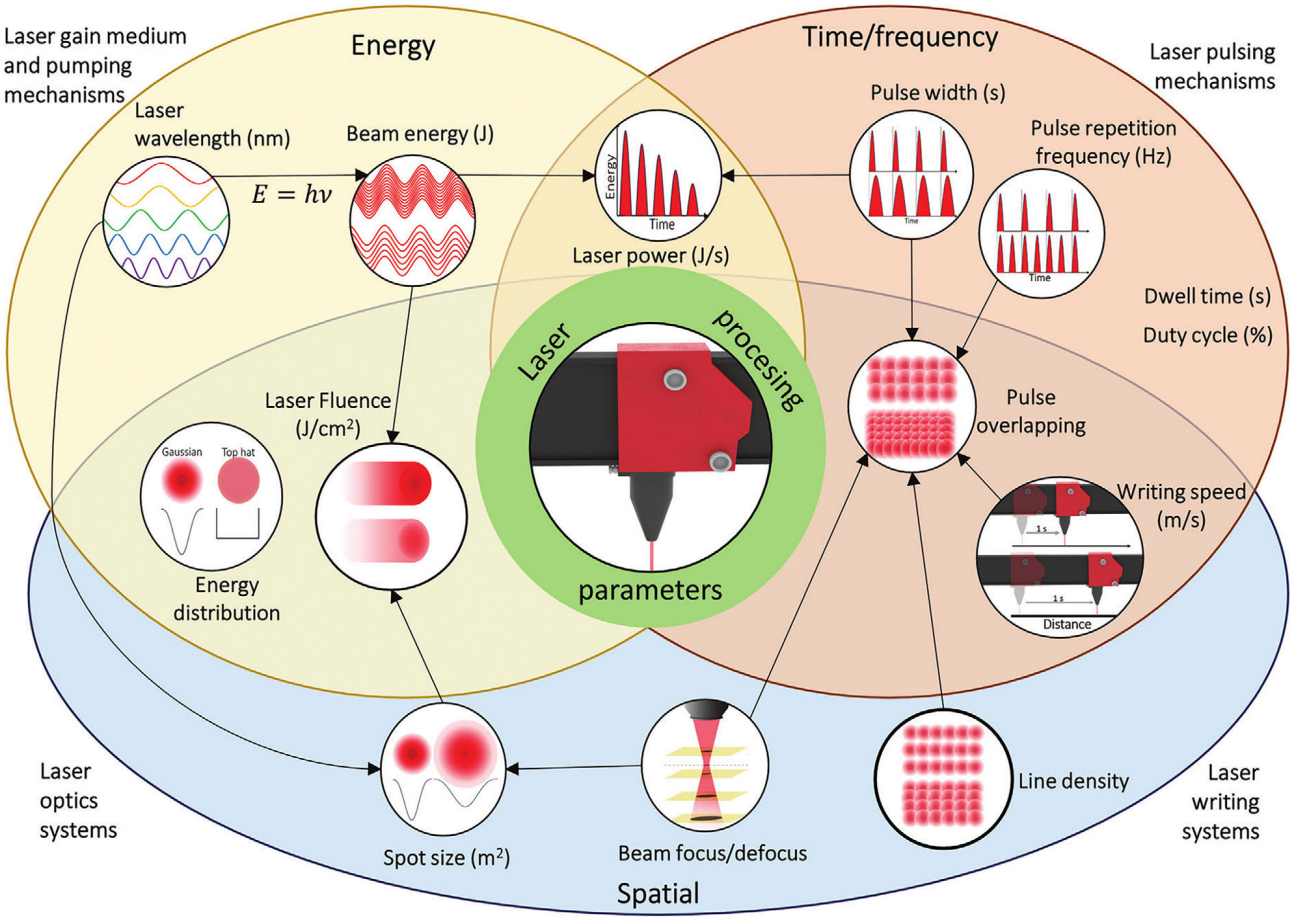
Scheme of laser fabrication parameters in the energy, time/frequency, and spatial domains and their interdependence. Reproduced under the terms of the CC‐BY 4.0 license.^[^ ^7^ ^]^ Copyright 2024, Pinheiro et al.

The lasing medium dictates the wavelength of the laser, typically ranging from deep ultraviolet (UV) for excimer lasers, up to far infrared (IR) for CO_2_ gas lasers (see **Figure** **3**), though both shorter and longer wavelengths are also possible. Other characteristics of the laser beam are dictated by the design of the laser cavity, including pulse length, pulse frequency, average and peak energy. Finally, the shaping optics, particularly the final focusing lens, dictate the size of the beam at the focal point (the beam waist), as well as the beam divergence away from the beam waist. These parameters are discussed below and summarized in **Figure** **4**.

*Wavelength, λ*, is the most intuitive parameter in terms of laser machining. In order for a laser beam to interact with a material, the material must be able to absorb photons of that light. This occurs through two primary mechanisms: electron and phonon excitation. Electron excitation typically occurs for wavelengths in the visible and near‐visible range (around 300 nm to 1500 nm), while phonon excitation occurs at longer wavelengths (in laser machining this is normally the 10.6 µm emission line of CO_2_ lasers). An exception to this occurs when very short laser pulse lengths are used, discussed below.

The relationship between wavelength and material absorption can be seen in Figure 3, which also shows various laser wavelengths. While CO_2_ lasers remain the most widely used laser in micromachining due to their low cost, the solid state Nd:YAG and Nd:YVO_4_ lasers, with fundamentals around 1060 nm, commonly also used at their second and third harmonics (≈530 and ≈355 nm, respectively) have become increasingly popular owing to their improved materials selectivity and superior spatial resolution. It is also worth noting that, while the absorption of both metals and polymers drops through the visible spectrum, this is due to quite different reasons: for polymer materials absorption drops because light transmission increases as the bandgaps of the materials are greater than the photon energy, while for metals the absorption drops as the reflectivity increases. A comparative overview of different wavelengths can be found in **Table** **1**.

**Table 1:** Comparison of the key aspects of different laser wavelengths.

Wavelength range	Polymer absorption	Metal absorption	Resolution limit*	Advantages	Limitations
**UV** **(<400 nm)**	Strong	Medium	<1.5 µm	Excellent for polymer ablation	High cost optics, Lower processing speed
**Visible to near IR** **(400–1100 nm)**	Variable, material dependent	Medium‐low	1.5–4.2 µm	Wide range of sources Good for thin‐film metal patterning	Inefficient for polymer processing
**Deep IR** **(>1100 nm)**	Variable, material dependent	Medium‐low (reflectivity dominates)	>4.2 µm (40.5 µm @ λ = 10.6 µm)	Rapid processing Good for cutting	Low resolution, High thermal damage

Note: * for a laser using a galvo system with F theta lens with typical values: *d*_0_ = 10 mm, *f* = 100 mm, *M* ^2^ = 1.2.

*Pulse length*, *t*_p_, is a critical parameter, particularly in modern laser machining. Pulse lengths typically vary from around 10 fs up to 10 µs or continuous wave (CW) lasers. Within this range, we can consider three distinct working regions: long pulse lasers, with pulse lengths over 1 µs; short pulse lasers with pulse lengths between 1 ps and 1 µs; and ultrashort‐pulse lasers, with pulse lengths below 1 ps.

Long pulse laser machining is dominated by thermal effects (melting and sublimation) due to the long durations allowing for thermal transfer. While useful for applications such as laser welding^[^ ^28^ ^]^ (Coles et al., manuscript under review) and cutting of thick materials, this also leads to large amounts of material melt and heat‐affected zones (Figure 2B). One common application of such long pulse lengths in bioelectronics is the creation of conductive carbon materials,^[^ ^29^, ^30^ ^]^ discussed further below.

Short‐pulse laser machining reduces the level of thermal effects as there is less time for thermal transfer to occur, promoting the occurrence of photochemical effects by photoionizing the material. Practically, many uses of short pulse lasers lead to a combination of thermal and photochemical effects.

Ultrashort‐pulse lasers almost entirely preclude the occurrence of thermal effects as electron–phonon coupling becomes negligible at these short timescales. In addition, the very high photon flux during these pulses leads to multiphoton absorption (MPA), allowing materials normally transparent to that wavelength to start absorbing. In this case, the energy of two or more photons that arrive at an atom simultaneously is combined to overcome the energy bandgap in the material. This MPA is heavily dependent on the likelihood of multiple photons hitting an atom simultaneously; as the probability of this is proportional to the square of the laser intensity, the multiphoton interaction volume falls off as 1/*I* ^2^, leading to a tightly confined voxel around the focus of the beam. Comparison of these different pulse length regimes can be seen in **Table** **2**.

**Table 2:** Comparison of the key aspects of laser pulse length. The characteristics of ablation threshold, HAZ size, and ablation characteristics are drawn from the discussion in Section 2.1, and the more extensive coverage of these in the textbooks from Bäuerle^[^ ^17^ ^]^ and from Dahotre and Harimkar.^[^ ^18^ ^]^

Pulse length	Ablation threshold	HAZ size	Ablation characteristics	Cost
CW* – µs	High	Large	Melting/boiling Melt ejection Photochemical breakdown	Low
**ns**	Moderate	Moderate (10s of µm)	Melting/boiling Melt ejection Photochemical breakdown Stress‐induced explosion	Medium
**ps**	Low	Small (≈µm)	Direct evaporation Some melt possible Explosive ablation	High
**fs**	Lowest	Negligible (<µm)	Direct evaporation No debris	Very high

Note: *CW = continuous wave.

*Pulse frequency* is another factor that influences the level of thermal effects during processing. By using lower frequencies, there is a longer time for the dissipation of thermal buildup between pulses, helping to reduce thermal damage. However, this comes at the cost of processing speed and quality as the laser beam has to be moved more slowly to achieve the same spot overlap.

The *peak* and *average energy* of the laser refer to the energy per pulse, and average energy of the beam, respectively. Depending on the mechanism of patterning and the pulse length, one or both of these parameters can be important in machining. In most materials, a minimum (threshold) energy density is required to initiate processing. For thermally dominated processes, the key parameter is the total energy received at the workpiece, so the average energy is key. However, for photochemical processes and particularly when MPA becomes a factor, the energy of each pulse is key. Both of these energies must also be considered with respect to the laser spot size, as it is the energy density (energy per area) that governs both of these regimes.*

*This is a consideration that is very rarely discussed in literature when reporting laser parameters. While the laser energy used may be reported, the laser spot size rarely is, making it impossible to meaningfully compare the energy densities used in different works, or to use this as a starting point for one's own work.

The *laser spot diameter*, ⌀, at the workpiece is the final, critical, parameter. The size of the laser spot determines resolution of the system, and the minimum feature size that can be achieved, and can be calculated as

(1) $$d_{f}=M^{2}\frac{\lambda f}{\pi d_{0}}$$

where *d*_f_ is the diameter of the beam at focus, M*M* ^2^ is the dimensionless beam quality factor describing the deviation from an idealized Gaussian beam,^[^ ^31^ ^]^ λ is the wavelength, *f* is the focal distance of the focusing lens, and *d*_0_ is the diameter of the laser beam before the focusing lens. It is clear that the spot size is directly proportional to the laser wavelength, and many high‐resolution machines rely on short wavelengths to achieve small spot sizes. Spot size can also be reduced by using a longer focal length and/or a larger aperture (to increase *d*_0_), collectively known as using a lens with a large numerical aperture, NA (NA = *d*_0_/*f* for a laser beam). The NA value of the lens also controls the divergence of the beam, or how quickly it spreads after the focal point, which can be an important consideration when trying to remove one layer of material without affecting subsequent layers. Finally, the distinction here between system resolution and minimum feature size that can be achieved should be noted. The *resolution*, defined by the spot size in Equation (1), is an intrinsic property of the laser and its optical configuration, representing the theoretical smallest feature that can be produced. In practice, however, the minimum feature size also depends on the thermal and optical penetration depth of the laser within the specific material, the extent of photothermal and photochemical reactions, and the overall cohesiveness of the material system.

### 2.3 Laser Machining Systems

A key aspect of laser machining systems is the manner in which the laser beam is directed to the sample. There are three main ways through which this can be achieved: movement of the laser writing head, movement of the sample, and deflection of the laser beam (Figure 2C–E), respectively, summarised in **Table** **3**.

Basic laser machining systems rely on movement of the writing head to create the desired pattern. In these systems the laser beam is reflected by a series of fixed mirrors into the writing head and then down through a focusing lens to the sample. The writing head moves in the XY plane to create the pattern and typically the sample is moved up and down in the Z plane to place it at the focal point of the beam. These systems are simple and inexpensive, but cumbersome and relatively slow. They are most commonly used with CO_2_ lasers, finding wide use in bioelectronics applications, particularly the creation of laser‐induced graphene (LIG).

An alternative is to directly move the sample, or workpiece, using a translation stage, underneath a fixed laser head. This reduces vibrations in the optical components by keeping them static, allowing for finer patterning of the sample.

Both write head and translation stage motion‐based systems are relatively slow, typically running in the 0.1–100 mm s^−1^ range, owing to the need to move heavy system components. They also commonly suffer low acceleration profiles, meaning the start/end of a cut is performed more slowly than the bulk, causing overexposure in these areas. While there are techniques to overcome this, including the use of lead‐ins/lead‐outs to accommodate the acceleration before machining starts, or varying the laser power during the acceleration phase, they can be challenging to implement satisfactorily. A significant advantage of these systems is the ability to process arbitrarily large workpieces, limited only by the range of the translation motors.

The third option is the use of deflection mirrors to steer the laser beam onto the sample, typically galvanometrically controlled. Due to the low weight of these mirrors, and rapid response of the galvanometric actuators, these systems allow very fast write speeds, up to 10 000 mm s^−1^. This is particularly important when rastering the laser beam over a large area, rather than simply following an outline, as it significantly reduces processing times. This approach also helps with some of the challenges associated with ultrashort‐pulse and high frequency lasers, particularly when processing a large area.^[^ ^32^ ^]^ However, when deflected away from the normal, the beam becomes distorted, which must be compensated for, either in the optical setup (typically using an F‐Theta lens) or by using a complex computer algorithm to vary the focal position of the beam. This need for a flat scanning field significantly reduces the area that can be processed by the laser, often leading to the need to combine a translation stage and a galvanometric system in a single machine.

One additional consideration when looking at beam delivery is the reflectivity of the work piece. For highly reflective samples such as gold or platinum thin films, normal‐incidence laser writing using either write‐head or process‐bed motion, poses a high risk of back‐reflection into the optics, potentially causing significant damage. Beam deflection, in contrast, mitigates this risk by angling the beam away from the normal, eliminating the possibility of back‐reflection. Consequently, machining of highly reflective materials is best performed using systems with beam deflection 1.

**Table 3:** Comparison of the key aspects of the three beam delivery systems described. Information gathered from a range of industrial publications and technical specifications.^[^ ^33^, ^34^, ^35^, ^36^, ^37^, ^38^ ^]^

Writing system	Speed	Accuracy	Process area	Cost
**Head motion** **(Gantry)**	Moderate (cm s^−1^)	10–100 µm	Very large >50 × 50 cm	Low
**Sample motion** **(XY stage)**	Low (mm s^−1^)	<µm possible	Large 10 × 10 to 100 × 100 cm	Medium
**Galvo**	Very High (m s^−1^)	Few µm	Small 10 × 10 to 100 × 100 mm	High

## 3 Laser Machining versus Alternative Fabrication Approaches

**Figure 5:**
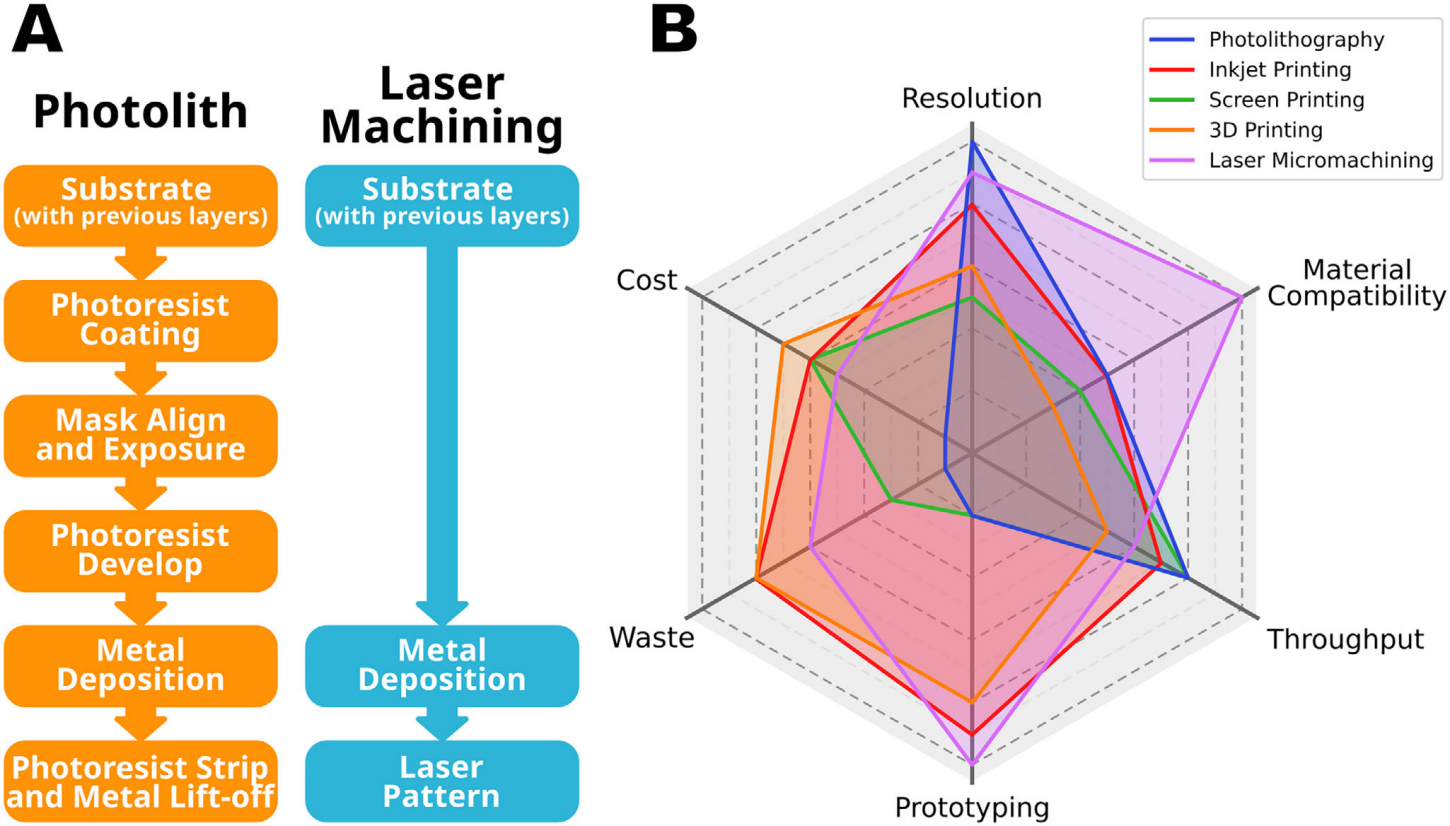
Comparison of fabrication methods. A) Typical process flows for fabricating a single metal layer through photolithography and through laser machining. B) Spider plot of the relative advantages of laser machining, photolithography, and different printing techniques. Values closer to the edge represent superior performance and are synthesized from several published reviews on flexible microelectronics and microfluidics fabrication,^[^ ^7^, ^40^, ^41^, ^42^, ^43^, ^44^ ^]^ along with the authors’ experience of these techniques. Exact performance will vary between different hardware and materials systems.

Laser micromachining has several notable advantages over alternative fabrication routes for bioelectronics, particularly the dominant approach of photolithography. The relative merits of several fabrication approaches are summarized in **Figure** **5**B based on our experience of them.

An immediate advantage is the cost of equipment and consumables. Laser machining tools are considerably less costly than the suite of equipment required for standard photolithography, commonly in the range of 5%–50% of the cost. In addition, far fewer consumables are required since photoresists, developers, and strippers are not used, and photomasks are redundant. While printing techniques offer similar cost advantages, this is offset by limitations in feature size and materials compatibility, and the need for processing chemicals.

Time is a second clear benefit of laser machining. Time savings come from removing the need for photomasks in the process, as well as the processing time itself. Since laser machining is a digital process, a CAD files is all that need be supplied to the machine, so there is no time lost waiting for new photomasks. This is particularly beneficial at the device prototyping stage, where small design changes can be implemented in a matter of minutes rather than days or even weeks. Inkjet and 3D printing similarly have this advantage, making them popular prototyping choices, though they lack the finesse and materials range required for high resolution device manufacture. The process itself, and the reduced number of processing steps compared to photolithography also save considerable time. Where photolithography may require five or more separate steps for a single layer, laser machining requires just two (Figure 5A). In addition, the laser machining itself can be rapid (depending on the type of system used), with many machines comfortably able to process thin metal layers in excess of 0.5 cm^2^ s^−1^, meaning a 100 mm diameter wafer may take on the order of a few minutes to entirely process if using a suitable machine. One caveat here is the relationship between laser spot size and speed: high speed machining is best achieved with a larger laser spot size, so more material is removed in a single scan line; however, a large spot size means larger feature sizes, limiting the miniaturization of devices. Nonetheless, even using a 2 µm laser spot size (the smallest size seen in this review), a 100 mm wafer could be expected to take some tens of minutes, similar to or better than the equivalent in photolithography. This processing speed, however, presents one of the challenges associated with laser‐based manufacturing, particularly when looking at volume production—because laser machining is an entirely serial process, the process time scales linearly with the number of wafers being processed, unlike photolithography which can take advantage of efficient batch processing at larger volumes to achieve a higher throughput at scale. Additive manufacturing techniques also suffer from this serial processing limitation, reducing their uptake for general production purposes.

The major limitation of laser machining, compared to photolithography, is resolution, or more precisely the minimum achievable feature sizes. In general, ablative laser machining is limited by the laser spot size [Equation (1)], with spot sizes below 1 µm hard to achieve and often leading to rapid deterioration of the optics owning to the very laser energy density created. In contrast, deep‐UV and nanoimprint lithography is able to achieve minimum feature sizes well below 1 µm, down to just a few tens of nanometers. On the other hand, laser machining offers significantly smaller feature sizes than can be achieved with most printing techniques (generally tens to hundreds of microns), with only two‐photon polymerization, itself based on laser processing, able to achieve the same sizes.^[^ ^39^ ^]^

Finally, the range of materials that can be processed offers a distinct advantage in the research environment. While printing is only possible with a limited range of materials (suitable inks, photopolymers, and filaments, with further limitations when printing multiple sequential layers), and photolithography can only be performed on materials compatible with the necessary resists, solvents, and etchants, laser machining can be performed on almost any material given the appropriate selection of laser wavelength and pulse duration.

While laser machining is not ideal for all applications, particularly high resolution or high throughput situations, it does represent a highly versatile approach to rapidly developing new bioelectronic devices at a price point well below photolithography and with feature sizes unachievable through printing techniques.

## 4 Laser Machining in Bioelectronics—Materials and Selected Structures

There are many different applications of laser machining in bioelectronics. The following section attempts to summarize many of these, providing both overviews of general themes, and specific examples of particularly interesting applications. As will be seen, the use of laser machining in bioelectronics has expanded greatly, particularly over the last five years. This expansion is driven by a variety of factors which has made these laser machining systems more available to researchers. In particular, the availability of high precision, high speed, short pulse laser systems increased through the 2010s, while the 2020s is seeing increased availability of ultrashort pulse systems, each of which has led to increases in machining resolution and the variety of processable materials, while reducing the unintended damage to polymeric substrates. For more detailed discussion of the evolution of laser micromachining and its use in microelectronics, the reader is directed to reviews by Swenson et al.,^[^ ^45^ ^]^ Rahim and Mian,^[^ ^46^ ^]^ Pinhero et al.,^[^ ^7^ ^]^ and specifically for ultrashort pulse lasers, Philips et al.,^[^ ^47^ ^]^ and Guo et al.,^[^ ^48^ ^]^ along with a recent, high‐level overview of the field in the book chapter from Bansal et al.^[^ ^49^ ^]^

### 4.1 Laser Cutting of Metal Foils and Silicones

Much of the early use of laser machining for bioelectronics took place in the mid‐2000s, using nanosecond lasers to cut metal foils (particularly Pt and PtIr) and silicone substrate/insulation layers, focused primarily at the universities of Freiburg (Germany), and New South Wales and Newcastle (Australia).^[^ ^50^, ^51^, ^52^, ^53^, ^54^, ^55^ ^]^

The first example of this came from the Suaning group at The University of Newcastle, Australia, who used a 532 nm (frequency doubled Nd:YAG) nanosecond laser to cut metal foils and silicone layers to create a 19‐channel microelectrode array.^[^ ^50^ ^]^ Their process involved several laser patterning steps, first to cut the metal foil (18 µm foils of either Pt or Al laminated onto a 20 µm silicone layer), then opening holes in a top 20 µm silicone layer to expose the electrode sites, and finally cutting through both layers of silicone to define the overall shape of the device. This processing was aided by a stream of nitrogen gas which cooled the samples, provided an inert processing environment, and prevented redeposition of ablated debris. After cutting the metal foils, excess material was simply peeled off the silicone by hand, leaving the desired patterns.

This process was quickly adopted by the groups of Lovell at the University of New South Wales, and Stieglitz at the University of Freiburg, both of whom used 1064 nm nanosecond laser systems to create an array of neural interfacing devices including retinal implants,^[^ ^56^, ^57^, ^58^ ^]^ cortical recording and stimulation arrays,^[^ ^52^, ^54^, ^55^, ^59^, ^60^ ^]^ and nerve interfacing devices,^[^ ^53^, ^61^ ^]^ as well as exploring the scaling limitations of laser fabrication,^[^ ^53^ ^]^ and the cytotoxicity of laser processed materials.^[^ ^62^ ^]^ This approach to device fabrication was also commercialized by several of these same researchers in the form of the neurotechnology company CorTec GmbH, based in Freiburg, who have gone on to develop a range of neural interfacing technologies based on laser patterned silicone and metal foils.^[^ ^63^, ^64^, ^65^, ^66^, ^67^ ^]^

### 4.2 Laser Patterning of Conducting Materials

A natural evolution of the earlier laser machining work is the introduction of a wider set of metals to be laser processed. This covers both a plethora of elements (and alloys) and a range of formfactors. Here, we distinguish between metal foils (generally above 1 µm in thickness and purchased as freestanding materials) and metal thin films (generally between 10 and 500 nm and deposited “in house” through a variety of means).

#### 4.2.1 Metal Foils

Due to the time required to ablate metals, most works showing laser patterning of metal foils follow a similar approach to the original work of Suaning.^[^ ^50^ ^]^ That is, the outline of the desired pattern is laser cut into the foil, and the remaining waste metal is manually removed from the device.

**Figure 6:**
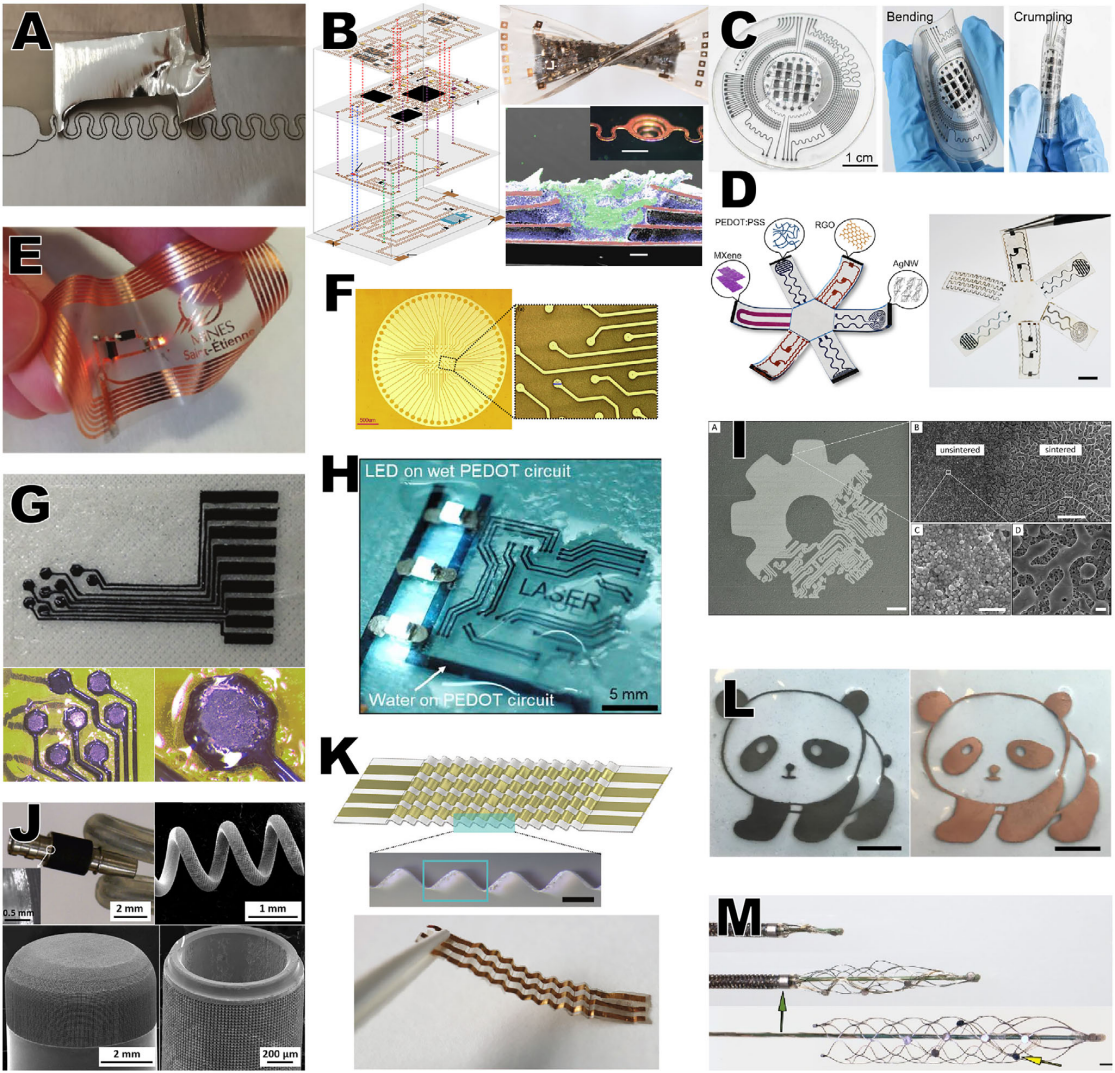
Examples of laser micromachining of conductive materials for bioelectronics. A) Cutting and peeling aluminum foil. Reproduced under the terms of the CC‐BY 4.0 license.^[^ ^86^ ^]^ Copyright 2018, Marchiori et al. B) Patterned copper foil with laser cut vias. Reproduced with permission.^[^ ^70^ ^]^ Copyright 2022, Springer Nature. C) Laser patterned MXenes. Reproduced with permission.^[^ ^87^ ^]^ Copyright 2024, The American Association for the Advancement of Science. D) Multimaterial laser patterning. Reproduced under the terms of the CC‐BY 4.0 license.^[^ ^88^ ^]^ Copyright 2024, Zhang et al. E) Laser patterned copper foil. Reproduced with permission.^[^ ^71^ ^]^ Copyright 2019, Wiley. F) Laser patterned thin‐film gold. Reproduced with permission.^[^ ^89^ ^]^ Copyright 2010, Elsevier. G) Laser cut conductive elastomer. Reproduced with permission.^[^ ^90^ ^]^ Copyright 2013, Royal Society of Chemistry. H) Laser‐stabilized PEDOT:PSS. Reproduced under the terms of the CC‐BY 4.0 license.^[^ ^91^ ^]^ Copyright 2022, Won et al. I) Laser sintered liquid metal. Reproduced with permission.^[^ ^92^ ^]^ Copyright 2019, American Chemical Society. J) High surface area platinum through laser surface processing. Reproduced under the terms of the CC‐BY 4.0 license.^[^ ^93^ ^]^ Copyright 2022, Amini et al. K) Stretchable interconnects through laser pattered origami structure. Reproduced under the terms of the CC‐BY 4.0 license.^[^ ^94^ ^]^ Copyright 2022, Del Dulca et al. L) Electroless plating of copper on laser activated polymer. Reproduced with permission.^[^ ^95^ ^]^ Copyright 2022, American Chemical Society. M) Laser cut stentrodes. Reproduced with permission.^[^ ^96^ ^]^ Copyright 2016, Springer Nature.

Copper foils were introduced as a laser machined component in devices by the Rogers group at Northwestern University, USA in 2017, who used an infrared laser (λ = 790–820 nm) to cut flexible metal tracks from copper foils ranging in thickness from 15 to 75 µm.^[^ ^68^ ^]^ However, this work did not show any direct application in bioelectronics. The same year, Tanabee et al. laser cut 25 µm copper foils to act as the receiving element of a wireless power transfer system for implanted, wireless nerve stimulation.^[^ ^69^ ^]^ The following year, Huang et al. used a 1064 nm, microsecond pulse laser to pattern 20 µm Cu foils into stretchable interconnects for a wearable motion tracking microelectronic device (**Figure** **6**B).^[^ ^70^ ^]^ This was followed in 2019 by work from de Mulatier et al. who used another 1064 nm laser system, this time with nanosecond pulse length, to laser cut a 5 µm copper foil for use as a wearable cardiac monitoring device (Figure 6E).^[^ ^71^ ^]^

While copper foil remains a relatively uncommon material in bioelectronics, largely due to its tendency to corrode unless well encapsulated, its use continues in laser fabricated wearable devices, including wearable display,^[^ ^72^ ^]^ sensing,^[^ ^73^, ^74^ ^]^ and power harvesting applications.^[^ ^75^ ^]^ One recent outlier is the work of Kim et al. who used a 1064 nm laser to cut 10 µm Cu foil, coated with a thin layer of gold and embedded in a silicone matrix, as a soft, compliant, peripheral nerve interface.^[^ ^76^ ^]^ Another is the recent work from Mariello et al., in which a 1064 nm, nanosecond laser was used to cut 18 µm copper foils, actively cutting around a curved surface, to create an inductively powered smart contact lens.^[^ ^77^ ^]^ In addition, patterning Cu foils to create flexible circuit boards (fPCBs) has become a standard part of the work from the Rogers group. They regularly use a 355 nm, picosecond laser specifically designed for fPCB manufacture (the Protolaser U4 from LPKF) to create either single or double sided fPCBs with a polyimide core, used for a wide variety of wearable and implantable applications (with suitable polymeric encapsulation),^[^ ^78^, ^79^, ^80^, ^81^, ^82^, ^83^, ^84^ ^]^ a process which is now being adopted by other groups.^[^ ^85^ ^]^

A number of other metal foils have been demonstrated as laser machined components for bioelectronic applications, though only a limited number of reports on these can be found.

In 2014, Mueller et al. introduced the nickel‐cobalt alloy, MP35N, as an alternative to Pt or PtIr foils for intrafascicular electrodes, showing both cutting and thinning of the 25 µm foils using a 355 nm picosecond laser system.^[^ ^97^ ^]^ In 2018, Chapman et al. similarly used stainless steel foils as an alternative to Pt, cut and surface patterned using a 1064 nm laser system for peripheral nerve interfacing.^[^ ^98^ ^]^

In 2018, Marchiori et al. used a 1064 nm, nanosecond laser to cut serpentine interconnects in 50 µm aluminum foils to act as effective stretchable interconnects between OECTs (Figure 6A).^[^ ^86^ ^]^ To aid in the removal of the waste material, a thermal release layer was used which could be manually debonded or heated with an additional laser pass to initiate removal. In 2024, Jeong et al. used 1 µm commercial gold leaf, along with commercial polyimide films, patterned with a relatively inexpensive long pulse UV laser system to demonstrate low cost fabrication of a neural interfacing device that was capable of detecting single unit activity from an ex vivo retinal ganglion cell culture.^[^ ^99^ ^]^ And in 2025 Wang et al. used a 355 nm, picosecond laser to cut 10 µm titanium foils for the fabrication of soft spinal cord stimulation paddles.^[^ ^100^ ^]^

#### 4.2.2 Transient Metal Films and Foils

Beyond the “standard” metal foils discussed above, laser processing also opens access to a wider range of more reactive metals normally considered unsuitable for bioelectronics due to fabrication challenges. These reactive metals rapidly break down in the body, as well as in the aqueous environments often experienced during standard photolithographic processes. While, by definition, devices fabricated from such materials do not last a long time in the body, they also do not need removing at the end of their useful lifetime, making them an interesting option for short term devices. For a broader overview of such materials, see the recent review from Zhang et al.^[^ ^101^ ^]^

Much of the work in this area has been carried out by the Rogers group, dating back to 2015 when they laser cut Mo foils to create a receiving coil for wireless power transfer to an implanted drug delivery device.^[^ ^102^ ^]^ Since then, that group has used laser cut foils of Mo, Mg, Fe, Zn, W, and MoO_3_ to produce a range of implantable, wireless devices, with the metal foils forming both the power transfer components^[^ ^103^, ^104^, ^105^, ^106^ ^]^ and the stimulating interfaces.^[^ ^12^, ^106^, ^107^, ^108^, ^109^ ^]^

Other groups have also used laser cut foils as part of their degradable devices, covering applications including dissolvable batteries,^[^ ^110^, ^111^, ^112^ ^]^ interconnects,^[^ ^113^ ^]^ and nerve stimulators.^[^ ^112^, ^114^, ^115^ ^]^

#### 4.2.3 Metal Thin Films

The natural evolution from patterning metal foils is the application of laser machining to thin metal films, ranging from 10 to 500 nm. These have been particularly attractive in bioelectronics over the last 20 years due to their much higher flexibility and compliance in comparison to metal foils, and the ability to process them using the wafer‐scale photolithography technology developed in the CMOS industry. This has allowed a transition to progressively smaller and more flexible devices, providing precision targeting of body tissue with reduced trauma.^[^ ^116^ ^]^ Here we look at the use of lasers to pattern thin films deposited on a substrate, generally by a physical vapor deposition process (sputtering, thermal evaporation, etc.).

Despite the advantages, laser machining of thin metal films was not seen in bioelectronics until 2009, when Hess et al. used a CO_2_ laser system to selectively ablate thin gold films on a PVA‐cellulose composite substrate to form single‐channel penetrating neural electrodes.^[^ ^117^ ^]^ The use of a CO_2_ laser here is particularly unusual owing to the high levels of thermal damage they cause. The following year Hayden and Dalton used an 800 nm, femtosecond laser system to pattern 200 nm thick gold on a glass carrier with features down to 20 µm, to fabricate a high‐density microelectrode array for lab‐on‐a‐chip applications, Figure 6F.^[^ ^89^ ^]^ Further development of thin metal film laser patterning was surprisingly slow throughout the 2010s, likely due to the superior spatial resolution achievable with more traditional photolithographic processing, the ready availability of these photolithography tools in research environments, and the thermal effects of the laser processing on the sensitive flexible substrates. Indeed, the next reported use of laser processed thin‐film metals that we can find was from Campana et al. in 2013 who used a 1064 nm laser system to pattern thin gold films as the source and drain electrodes on a PLGA substrate for an organic FET.^[^ ^118^ ^]^ This was followed a couple of years later by work from the Asplund group developing neural interfacing devices, where a 1064 nm, nanosecond laser system was used to selectively pattern their Ti/Pt/IrOx metallic layers.^[^ ^119^ ^]^

Since 2019 there has been a considerable rise in the use of laser micromachining to pattern thin‐film metals, likely due to increased availability of laser systems capable of ablating metals without significantly damaging the supporting material 2019 saw three different examples of laser machined thin‐films, from three different groups, with three quite different laser tools. She and Allen used a UV excimer laser to pattern gold films, as well as cut the polyimide substrate, as part of the fabrication of dissolved oxygen sensors.^[^ ^120^ ^]^ Shim et al. used a 355 nm, nanosecond‐pulse laser system to define the metal tracks of a neural depth probe, along with a CO_2_ laser system to cut the cyclic olefin polymer substrate and encapsulation layers before thermal lamination.^[^ ^121^ ^]^ And Chae et al. used a 1064 nm, nanosecond laser system to define complex serpentine and kirigami type interconnect lines in gold thin‐films before cutting similar designs into the polyimide substrate and encapsulation layers to fabricate highly stretchable, body‐conformal electronics.^[^ ^122^ ^]^

The 2020s has seen considerably more publications featuring laser structuring of thin metal films, largely due to the proliferation of short and ultrashort pulse laser systems, in the visible and near IR wavelength ranges (λ ≈ 500–1100 nm), has given many more researchers the ability to micromachine these thin‐film metals without causing significant damage (particularly thermal) to the sensitive polymer substrates. These are broadly split between recording and stimulating electrodes for in vivo and in vitro applications,^[^ ^123^, ^124^, ^125^, ^126^, ^127^, ^128^, ^129^, ^130^, ^131^, ^132^, ^133^, ^134^, ^135^ ^]^ stretchable interconnects (Figure 6K),^[^ ^94^, ^136^, ^137^ ^]^ sensors (Figure 6D),^[^ ^88^, ^138^, ^139^, ^140^, ^141^, ^142^, ^143^, ^144^ ^]^ and stimulation devices.^[^ ^77^, ^145^ ^]^ Almost all of these works use nanosecond‐pulsed lasers, which are well suited to the structuring of metals as this time‐frame allows for effective thermalization and removal of metals without significant thermal transfer to the underlying polymer layers. On the other hand, the wavelengths used are evenly split between 1064 nm and near‐UV since the metals absorb strongly across these wavelengths. It is worth highlighting here some of these works that leverage the unique attributes of laser machining. For example, Lee et al. used their laser system to selectively ablate metallic lines in their devices postfabrication to hard code a unique address in each so as to create addressable devices for neural recording,^[^ ^127^ ^]^ an example of laser programmed memory that allows configurable hardcoding of data in a device.^[^ ^146^ ^]^ Another example is the ability to fabricate devices out‐of‐plane, thanks to the non‐contact nature of the laser machining and the ability to dynamically adjust the focal height. This is exemplified by recent works from both Bernhard Wolfrum's team, and our own work.^[^ ^77^, ^133^, ^134^ ^]^

#### 4.2.4 Metal Surface Patterning

In addition to cutting or ablating metal thin‐films and foils, there is a growing interest in using laser machining to enhance the surface properties of metal (and other materials). This so‐called laser surface processing is shown to cause physical, chemical, and electrochemical changes in the material surface, altering wettability, durability, adhesion properties, and charge storage. While the field of laser surface processing is too broad for this review, it is worth discussing the highlights with regard to metal (electrode) surfaces. For a broader discussion of laser surface processing, readers are directed to the recent reviews by Moskal et al. and Alsaigh.^[^ ^32^, ^147^ ^]^

As far back as 2008 it was shown that surface patterning of cochlear implant electrodes could effectively mediate cell growth, increasing neuronal attachment and decreasing fibroblast development.^[^ ^148^, ^149^ ^]^ Considerable work from the Lovell group, and others, in the early 2010s went on to show that laser surface structuring of platinum electrodes could effectively increase the surface area, thereby decreasing the electrical impedance^[^ ^58^, ^150^, ^151^, ^152^ ^]^ without affecting the biocompatibility of the materials used.^[^ ^10^ ^]^ Several different laser systems, with a wide range of wavelengths and pulse widths were used for this work, all having slightly different interactions with the metal surface, but all effectively increasing the surface area.

More recently it was found that ultrafast laser treatment (pulse lengths below a picosecond) goes beyond linear surface topology changes achieved by simply ablating material on the length scale of the beam width, additionally inducing surface structuring at significantly shorter length scales (Figure 6J). This phenomenon, referred to as laser‐induced periodic surface structuring (LIPSS), was first reported in a medical device by Kelly et al. in 2020. They used a 1030 nm, femtosecond laser to surface pattern a commercial PtIr microelectrode probe, dramatically increasing the charge injection capacity and decreasing the 1 kHz impedance of the probe tip by an order of magnitude compared to the unstructured tip.^[^ ^153^ ^]^ Since this first report on LIPSS in metal electrodes, the technique has been applied to a range of electrode surfaces, including neural electrodes,^[^ ^154^, ^155^, ^156^, ^157^, ^158^ ^]^ pacemaker leads,^[^ ^93^ ^]^ and stents^[^ ^159^ ^]^ to both improve the electrochemical performance, and mediate the biological reaction to the electrodes.

#### 4.2.5 Liquid Metals

Metals which remain liquid at room temperature are an attractive prospect in soft, conformal electronics due to their inherent flexibility and stretchability, and their self‐healing properties.^[^ ^160^, ^161^, ^162^, ^163^ ^]^ Laser machining has been used in two ways to fabricate bioelectronic devices based on liquid metals, as well as being used in the creation of the liquid metals themselves.^[^ ^164^, ^165^ ^]^ The first is laser patterning the substrate onto which the liquid metal will be deposited (normally a silicone elastomer) in order to confine the liquid metal to specific regions. This patterning can take the form of either engraving the substrate before filling with the liquid metal,^[^ ^166^ ^]^ or directly modulating the surface structure of the substrate to control the metal wettability using ultrashort pulse lasers.^[^ ^167^, ^168^ ^]^ This approach has been used to create several health tracking^[^ ^166^, ^169^, ^170^ ^]^ and sensing devices,^[^ ^167^, ^168^, ^171^ ^]^ as well as a cochlear implant electrode array.^[^ ^172^ ^]^ This surface structuring relies on lasers with wavelengths that interact strongly with the silicone substrate, either UV or deep IR (10.6 µm). The second approach involves directly patterning the liquid metals. This can be either directly ablating films of liquid metals, leaving behind the desired pattern, or effectively sintering liquid metal particles dispersed throughout an insulating matrix. Direct patterning has been demonstrated for several stretchable electronic^[^ ^173^, ^174^, ^175^ ^]^ and sensing applications,^[^ ^176^, ^177^ ^]^ relying again on UV lasers. Local activation (or sintering) of the liquid metals, where the laser provides thermal energy to break down both the insulating matrix material and the insulating metal oxide coating that forms rapidly on the surface of exposed liquid metals, remains a somewhat underdeveloped research area, with only a few papers demonstrating wearable sensor devices^[^ ^92^, ^178^, ^179^ ^]^ (Figure 6I), or more general applications of stretchable electronics.^[^ ^180^, ^181^ ^]^ While there is a growing body of work looking at laser processing of liquid metals,^[^ ^182^, ^183^ ^]^ applications in bioelectronics, particularly implanted devices, remains very limited, with concerns about leakage of the liquid and the biological impact of this being a significant hurdle.^[^ ^184^ ^]^

#### 4.2.6 Other Metal Patterning Processes

The previous section looks at subtractive patterning of metal thin‐films, analogous to traditional photolithography. However, the thermal nature of laser processing also allows for other, additive, approaches. One such approach is selective laser sintering, in which the thermal energy of the laser is used to locally heat nanoparticles which causes them to join together in a pseudo‐homogeneous layer, and which can be extended into 3D through spatial control of the laser focus. This approach has been well reviewed elsewhere^[^ ^7^, ^185^, ^186^ ^]^ and so is not covered here.

*Metallic Precursor Decomposition*: Another approach enabled by the thermal nature of laser processing is the photothermal decomposition of metallic precursors. Here, aqueous precursor solutions are coated onto a substrate, normally by spin‐coating, before being patterned using a laser system. As the laser light is absorbed by the precursor, it decomposes to leave a metallic layer only in the area where the laser has passed. To date, work demonstrating this approach has focused on wearable applications, mostly sensors, likely due to the chemicals involved in the metallic precursors and concerns around ensuring their complete removal. Nonetheless, recent work has shown wearable devices fabricated with Mo_3_C_2_,^[^ ^187^ ^]^ Ni,^[^ ^188^, ^189^ ^]^ CuRu,^[^ ^190^ ^]^ MoO_2_,^[^ ^191^ ^]^ and Co_3_O_4_^8^ as active materials, generally achieved using short wavelength (355 or 532 nm) and long‐pulse or continuous‐wave lasers. The long pulse times are a key aspect of this approach, where the longer time allows for efficient thermalization of the precursor, necessary to trigger the desired chemical decomposition.

*Nanowires and Nanoparticles*: A final solution‐processed route that has been explored with laser patterning is the use of metallic nanowires and nanoparticles. These can be coated onto a substrate and cured to form a continuous film, and then patterned much like the PVD deposited thin films above. As with photothermal reduction, most applications of this approach have been shown in wearable applications, with the majority focusing on silver nanowires (AgNWs). These have taken the form of body‐conformal electrodes^[^ ^192^, ^193^, ^194^ ^]^ and power transfer coils.^[^ ^195^, ^196^ ^]^ Interestingly, Zhang et al. did recently report an implanted sensor system using AgNWs, along with several other interesting materials, all patterned with a 1064 nm, nanosecond laser system.^[^ ^88^ ^]^

*Cutting Metallized Textiles*: For wearable applications, laser cutting provides an additional useful fabrication route: the direct cutting of metallized textiles. Here, a laser can be used to cut the metallized material before attaching it to a secondary textile substrate, and integrating with other components to create functional, wearable devices. This has recently been shown by Ramuz et al., for the fabrication of a haptic sensing glove, in which a CO_2_ laser was used to cut a commercial Ni/Cu coated conductive textile for interconnects,^[^ ^197^ ^]^ and by Luo et al., who used a similar conductive textile, along with liquid metals for VIAs and LIG sensors, to create a multifunctional health monitoring system.^[^ ^198^ ^]^

#### 4.2.7 Carbon Materials

One of the most widespread uses of laser fabrication in bioelectronics is the laser‐induced carbonization of polymers, most commonly polyimide. Variously referred to as laser pyrolysis, laser carbonization, or laser‐scribed graphene, this is most commonly known as LIG. First demonstrated in 1983 using a long pulse argon laser,^[^ ^199^ ^]^ there is now a huge body of work on LIG, with applications ranging from simple wearable resistive strain sensors through to fully implantable, active transistor devices. Beyond polyimide, LIG has now been demonstrated on a wide variety of synthetic, natural, and bio‐derived carbon‐rich materials, produced with a huge variety of laser systems from continuous wave CO_2_ lasers through to UV femtosecond lasers (though the former remains the dominant category for their availability and ease of use). Due to the breadth of this field, LIG‐based work is not covered further here and the reader is directed to one of the many comprehensive reviews of LIG in e‐skin biosensors,^[^ ^200^ ^]^ bioelectronics^[^ ^30^, ^201^, ^202^ ^]^ and more general electronics.^[^ ^203^, ^204^, ^205^, ^206^ ^]^

Beyond directly creating carbon allotropes on a substrate, lasers have also been used to pattern conductive carbon layers produced by other means. As with the nanowires discussed above, carbon conducting layers made of graphene, carbon nanotubes (CNTs), or simple carbon black, can be created through solution processing routes (commonly spin coating, blade coating, or inkjet printing), and then patterned by laser ablation to leave conductive structures. This simple approach has been used to fabricate neural electrodes,^[^ ^78^, ^88^, ^125^, ^207^ ^]^ OECTs,^[^ ^208^ ^]^ sensors,^[^ ^88^ ^]^ and for in vitro cell culturing applications,^[^ ^209^ ^]^ generally achieved with nanosecond lasers at a variety of wavelengths. These carbon materials can also be incorporated into a polymeric matrix before patterning to enhance their electrical and/or mechanical properties. For example, polydimethylsiloxane (PDMS) can be loaded with carbon materials and then laser patterned to create ultrasoft and stretchable devices.^[^ ^173^, ^210^, ^211^ ^]^ Alternatively, these carbons can be mixed into a conducting polymer such as PEDOT:PSS before laser patterning to enhance the film's electrical properties.^[^ ^212^, ^213^ ^]^

As well as patterning conductive layers, laser processing can also be used to enhance the intrinsic electrical properties of carbon materials. This is most commonly seen with the laser reduction of insulating graphene oxide to conducting reduced graphene oxide (rGO). This process has been used to fabricate wearable sensors,^[^ ^214^, ^215^, ^216^, ^217^ ^]^ and dry electrodes,^[^ ^218^ ^]^ and for improved neurite culturing,^[^ ^219^ ^]^ without the need to remove the precursor graphene oxide material. Laser treatment has also been shown to improve the conductivity of carbon nanotubes, which has been applied in the creation of electrically conductive bioscaffolds,^[^ ^220^ ^]^ and for wearable strain sensors.^[^ ^221^ ^]^

#### 4.2.8 PEDOT

The conducting polymer, poly(3,4‐ethylenedioxythiophene) (PEDOT), most commonly stabilized with polystyrene sulfonate (PSS), is now a stalwart of bioelectronic devices, widely used as the active material in sensors, and as electrode coating for improved electrochemical and mechanical properties. Laser patterning of PEDOT has been ongoing since 2001, with early demonstrations of laser patterned transistor devices,^[^ ^222^ ^]^ as well as exploration of ablation processes with multiple different laser systems able to pattern features down to around 20 µm.^[^ ^223^ ^]^ However, early work on PEDOT laser ablation was dominated by optoelectronic applications,^[^ ^224^, ^225^, ^226^, ^227^ ^]^ and a few transistor type applications,^[^ ^222^, ^228^, ^229^ ^]^ supported by work investigating the ablation dynamics directly.^[^ ^230^, ^231^, ^232^, ^233^, ^234^, ^235^, ^236^, ^237^ ^]^

It was not until the mid‐2010s that laser patterned PEDOT began to be considered for use in bioelectronic applications. In 2017 it was shown that an ultrashort‐pulse laser could be used to pattern a PEDOT:PSS film with a self‐assembled monolayer (SAM) of cell‐attracting or cell‐repelling material to control cell adhesion dynamics.^[^ ^238^ ^]^ The use of laser patterning here was essential since the SAMs are very fragile, and do not readily survive other patterning approaches. At the same time, another work showed direct patterning of PEDOT:PSS films (both full thickness ablation and partial thickness patterning) to control cell adhesion for cell‐on‐chip applications.^[^ ^239^ ^]^ Since then, a range of work has exploited laser patterning of PEDOT films to create bioelectronic devices

Recently there has been significant interest in the interaction of laser radiation with PEDOT:PSS films beyond film ablation. It has been shown that laser writing can be used to control film stability and adhesion to substrate,^[^ ^11^, ^91^, ^240^, ^241^, ^242^, ^243^, ^244^, ^245^, ^246^ ^]^ (Figure 6H), as well as selectively enhance the film conductivity.^[^ ^22^, ^247^, ^248^, ^249^, ^250^, ^251^ ^]^ These effects have been shown to be due to the introduction of thermal energy to the hydrated films which allows migration and agglomeration of the conducting PEDOT regions within the film.^[^ ^22^, ^91^, ^252^ ^]^ These effects have been exploited to create a range of devices, including OECTs,^[^ ^22^, ^245^ ^]^ sensors,^[^ ^240^, ^243^, ^251^ ^]^ neural interfaces,^[^ ^91^, ^242^, ^245^ ^]^ stretchable interconnects,^[^ ^11^ ^]^ thermoelectric devices,^[^ ^250^ ^]^ and both dry and wet electrophysiology electrodes.^[^ ^253^ ^]^ Most recently, this process has been shown to hold in PEDOT:PSS hydrogels in addition to pure PEDOT:PSS thin‐films, demonstrated as both nerve cuff electrodes,^[^ ^254^ ^]^ and as a triboelectric nanogenerator material embedded in sutures for tension monitoring.^[^ ^255^ ^]^ A more comprehensive discussion of this phenomenon can be found in the recent review from Zhu et al.^[^ ^256^ ^]^

Laser patterning has also recently been used to allow the patterning of other exotic forms of PEDOT including new hydrogel formulations,^[^ ^213^, ^257^, ^258^ ^]^ and aerogels,^[^ ^259^ ^]^ as well as conductive elastomers (Figure 6G). This last form of PEDOT, with PEDOT:PSS blended with polyurethane elastomer to create a flexible and stretchable conductive material, has shown great potential for bioelectronic applications in wearable,^[^ ^260^, ^261^ ^]^ and implantable electrodes for physiological monitoring,^[^ ^90^, ^262^, ^263^ ^]^ as well as controlled drug delivery.^[^ ^264^ ^]^ The processing of these new form factors of PEDOT is facilitated by laser machining as they are overly sensitive to more traditional approaches, and/or are otherwise incompatible with them. PEDOT:PSS has also been used as an amendment to LIG‐based devices, touched on above.

#### 4.2.9 MXenes

MXenes have emerged as a promising materials class for bioelectronics only in the last few years. As such, little work has explored the use of laser micromachining with them, though some is starting to emerge. As with other materials, such as PEDOT, MXenes can be laser processed in either pure, free‐standing forms, or integrated into a host material before shaping through laser machining. To date, the use of freestanding, laser‐processed MXene films have been limited to sensor applications, mostly for strain sensors (Figure 6C),^[^ ^87^, ^88^, ^265^ ^]^ while MXenes have also been combined with laser‐induced graphene for biopotential monitoring^[^ ^266^, ^267^ ^]^ and chemical sensing,^[^ ^268^ ^]^ and with reduced graphene oxide to create implantable power storage.^[^ ^269^ ^]^ A simple yet effective approach to direct patterning of MXenes was demonstrated by Driscoll et al., who used a CO_2_ laser system to create recesses in a cellulose substrate which they then filled with an MXene ink to create sensing and stimulation electrodes for both cutaneous and cortical applications.^[^ ^270^ ^]^

### 4.3 Laser Patterning of Insulating Polymers

#### 4.3.1 Parylene C

Alongside their work with metal foils and silicones, the Stieglitz group started introducing Parylene C as a support layer to improve the mechanical properties of their devices in 2011.^[^ ^60^ ^]^ Following from earlier work on Parylene C ablation, which used KrF excimer lasers at 248 nm to target the strong optical absorption of Parylene C below 250 nm,^[^ ^277^ ^]^ they showed that 15 µm Parylene C films could be successfully patterned using the same 1064 nm, nanosecond‐pulse laser used to cut their metal and silicone layers. Parylene C then became a staple part of their design architecture, patterned either as part of the stack^[^ ^278^, ^279^, ^280^, ^281^ ^]^ or as a freestanding film,^[^ ^60^ ^]^ using either their original 1064 nm laser, or later a 355 nm, picosecond replacement. Since then, many other groups have applied laser processing to Parylene C for multiple different applications and using many different laser systems. Rashid et al. used a CO_2_ laser cutter to cut through a gold/Parylene C/Teflon stack to create the source/drain contacts of a self‐aligned, PEDOT:PSS‐based OECT without requiring photolithographic processing.^[^ ^282^ ^]^ Sim et al. also used a CO_2_ laser to cut Parylene C as part of a stack with PDMS to act as a support substrate for flexible electronics,^[^ ^283^ ^]^ and Coles et al. used a similar approach to create a shape‐actuating ECoG device.^[^ ^28^ ^]^ Coles et al. have recently expanded on this concept and eliminated the PDMS layers by directly laser welding two layers of Parylene C to create a self‐contained fluidic chamber which they employed as the shape‐actuating component of a peripheral nerve cuff. (Coles et al., manuscript under review)

**Figure 7:**
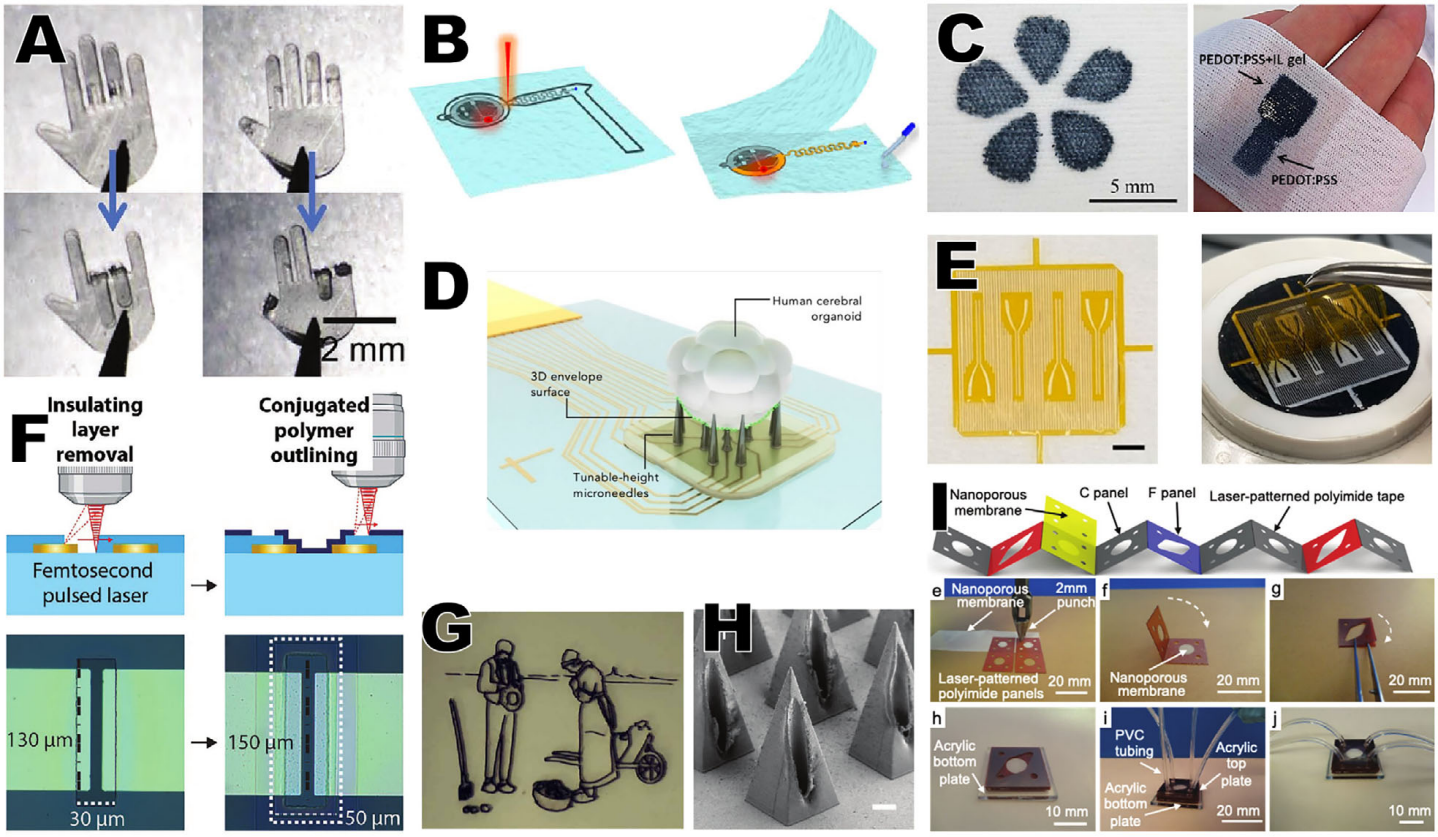
Examples of laser micromachining insulating materials for bioelectronics. A) Laser‐cut, self‐folding polymer. Reproduced under the terms of the CC‐BY 4.0 license.^[^ ^129^ ^]^ Copyright 2023, Hiendlmeier et al. B) Laser‐cut silicone encapsulation. Reproduced with permission.^[^ ^85^ ^]^ Copyright 2025, Springer Nature. C) Laser‐cut Kapton foil used as a deposition mask for PEDOT:PSS. Reproduced under the terms of the CC‐BY 4.0 license.^[^ ^271^ ^]^ Copyright 2015, Takamatsu et al. D) Direct laser milled microneedles for 3D organoid monitoring. Reproduced under the terms of the CC‐BY 4.0 license.^[^ ^77^ ^]^ Copyright 2023, Mariello et al. E) Laser‐cut Kapton mask used for biomass filtration. Reproduced under the terms of the CC‐BY 4.0 license.^[^ ^272^ ^]^ Copyright 2022, Ando et al. F) Laser milling of Parylene C without damage to the underlying gold. Reproduced under the terms of the CC‐BY 4.0 license.^[^ ^273^ ^]^ Copyright 2020, Nair et al. G) Direct laser writing of silicon carbide on silicone elastomer. Reproduced with permission.^[^ ^274^ ^]^ Copyright 2020, The American Association for the Advancement of Science. H) Laser milled microfluidic channels in pre‐formed microneedles. Reproduced with permission.^[^ ^275^ ^]^ Copyright 2023, Wiley. I) Laser milled microfluidic channels in stacked polyimide sheets. Reproduced with permission.^[^ ^276^ ^]^ Copyright 2021, Wiley.

Troughton et al. used another 1064 nm, nanosecond‐pulse system to cut out Parylene C‐based, implantable pulse oximeter devices,^[^ ^142^ ^]^ while the group of Bernhard Wolfrum regularly use a 355 nm, nanosecond laser system to outline Parylene C‐based devices (**Figure** **7**A).^[^ ^94^, ^129^, ^130^ ^]^ Li et al. also used a 355 nm laser for cutting Parylene C, though with a shorter 20 picosecond pulse width. With this they outlined a deep‐brain recording device, and opened electrode recording sites onto a sintered silver ink electrode site.^[^ ^284^ ^]^

A particularly interesting use of laser micromachining is the opening of recording sites on 3D fabricated electrodes that have been coated in Parylene C as an insulating layer. The concept of laser opening electrode tips coated with Parylene C has been around for more than two decades. Originally used to open the tips of Utah type electrode arrays through UV excimer laser processing,^[^ ^285^, ^286^ ^]^ this early work suffered significant challenges due to damage caused to the underlying device tips.^[^ ^287^, ^288^ ^]^ Though work continues on so‐called “deinsulation” of electrode arrays with excimer lasers,^[^ ^289^ ^]^ there has been a move towards looking at alternative approaches based on more modern laser systems. Brown et al. used a 1035 nm, femtosecond laser to remove the Parylene C passivation from the tip of their 3D printed and metallized electrodes to create a penetrating cortical electrode array.^[^ ^9^ ^]^ The Wolfrum group have also used their 355 nm, nanosecond laser to open the tips of 3D printed nano‐pillars with Parylene C encapsulation for use in organoid recordings.^[^ ^133^, ^134^ ^]^

It is worth discussing here some of the challenges in laser processing of Parylene C. Above 282 nm, the optical absorption of Parylene C is very low, meaning at these wavelengths only a small fraction of incident laser energy is able to affect the material. In many of the applications discussed, this is overcome by simply increasing the incident power until cutting is achieved. This is only possible when the Parylene C is cut along with all the other layers and there is no other material below the Parylene C, or through clever positioning of the target area to minimize the impact of this high energy beam.^[^ ^133^, ^134^ ^]^ Alternatively, as with much of the early laser deinsulation studies, short wavelengths can be used which are more readily absorbed by the Parylene C. A final strategy is the use of ultrashort‐pulse lasers, which push the interaction process into a multiphoton absorption (MPA) regime, thereby removing the limitations of “normal” optical absorption.

The Zeglio group, in Stockholm, has also shown some recent work removing Parylene C passivation from a conducting (gold) layer using a femtosecond laser system for the creation of OECT‐based sensors (Figure 7F).^[^ ^273^, ^290^ ^]^ This work uses the laser of a Nanoscribe system, normally used for high‐precision 3D printing, with a wavelength of 780 nm, and successfully shows the removal of Parylene C from gold source–drain contacts to create OECT devices (albeit very slowly, working at less than 1 mm^2^ h^−1^ to remove around 1 µm thickness of Parylene C). The integrity of the thin (100 nm) gold layer is not explicitly discussed, but it can be surmised that a significant level of MPA is occurring here in order to successfully remove the Parylene C layer while preserving the gold layer.

#### 4.3.2 Polyimide

Laser processing of polyimide has been under investigation for over 40 years, starting from the early 1980s,^[^ ^291^ ^]^ continuing throughout the late 20th and early 21st century, largely concerned with excimer (λ ≈ 150–350 nm) and CO_2_ (λ = 10.6 µm) laser approaches for ablation and pyrolysis (see section on carbons above).^[^ ^25^, ^199^, ^292^, ^293^, ^294^ ^]^ The turn of the millennium saw the transfer of this technology into the realm of bioelectronic applications in the form of microfluidic biosensor chips fabricated in polyimide and structured through laser processing.^[^ ^295^, ^296^, ^297^, ^298^, ^299^, ^300^ ^]^ It was not until the 2010s that laser structured polyimide started to appear in implantable bioelectronic devices. In 2012 Ismailova et al. used a CO_2_ laser to scribe the outline of a neural probe, simultaneously cutting both the polyimide substrate and an SU‐8 encapsulation layer.^[^ ^301^ ^]^ This was followed by further work from the Malliaras group developing cutaneous electrodes for several electrophysiology applications based on laser cut commercial polyimide sheets (Kapton), cut using a 1064 nm, nanosecond‐pulse laser system.^[^ ^302^, ^303^, ^304^ ^]^ The Rogers group also started to introduce laser cutting of polyimide films for implantable devices in the following years, including for creating stretchable interconnects,^[^ ^305^, ^306^ ^]^ optogenetic devices,^[^ ^307^ ^]^ and cardiac monitoring applications.^[^ ^308^ ^]^ While the information on the laser systems used by Rogers is sparse, it is mentioned that both UV (355 nm) and IR (790–820 nm) lasers were used for these works.

It is interesting to note here that, in all of these applications, the laser systems are used only to cut and shape the polyimide layers, with photolithography used to pattern the conducting layers that also make up the devices. This is a significant contrast to later works in which laser systems are used to pattern conducting layers in addition to the polymer substrate, and particularly curious given that both the Malliaras and Rogers groups use laser systems from LPKF which are specifically designed to perform large area patterning of devices (originally aimed at PCB prototyping).

Following the work of the Malliaras and Rogers groups, the late 2010s and the 2020s have seen a marked increase in the use of laser processed polyimide for bioelectronic applications. Recently there have been multiple examples of laser machining being used to both pattern thin metal layers and define the shape of devices, primarily for either neural interfacing or sensor applications.^[^ ^88^, ^120^, ^122^, ^128^, ^141^ ^]^

A convenient alternative use of laser cut polyimide sheets is as a mask to aid in the deposition of other layers on a substrate. Takamatsu et al. used this approach to selectively deposit PDMS on a textile substrate before adding a conductive polymer (PEDOT:PSS) for wearable electrocardiography electrodes^[^ ^271^ ^]^ (Figure 7C), while Huang et al. used a laser cut polyimide film as a shadow mask for depositing Mg to create bioabsorbable electronics.^[^ ^309^ ^]^ Ando et al. used a laser cut polyimide mask as a filtration mask for filtration deposition of nanofibers to create a super flexible nerve cuff based on novel biopolymer fibers, Figure 7E.^[^ ^272^ ^]^ Most recently, Tao et al. showed the use of both polyimide sheets, cut with a CO_2_ laser for low‐resolution patterning, and Parylene C sheets, cut with a 780 nm femtosecond laser system for sub‐micrometer features. These were used to pattern PEDOT:PSS and Pt thin films in the fabrication of neural recording probes.^[^ ^310^ ^]^ Here, laser cutting provides a very cheap and quick approach to creating masks, allowing rapid prototyping at minimal cost.

#### 4.3.3 Silicones

Silicone elastomers, particularly PDMS, have been a major component in many, if not the majority of bioelectronic devices of the 21st century. As a soft, stretchable, highly biocompatible material, they have formed the substrate and encapsulation materials for a wide range of devices, with their liquid precursor state and low curing temperature making them ideal for functionalizing with a wide variety of amendments.^[^ ^311^, ^312^, ^313^ ^]^

As discussed above, the early laser‐based work from the Lovell and Stieglitz was heavily reliant on laser cutting of silicone layers, alongside metal foils, to create a wide range of neural interfacing devices. Initially based on cutting freestanding foils of silicone before laminating together, this later evolved into being able to open holes directly onto the metal layers. Many other groups have continued to use this approach across a range of neural interfacing applications.^[^ ^73^, ^97^, ^98^, ^314^, ^315^, ^316^ ^]^

However, directly cutting through silicone layers is not the only way in which laser micromachining has been used to create silicone‐based bioelectronic devices, and indeed laser cutting pure silicone materials remains somewhat challenging, particularly without damaging the layers below. As alluded to above, it is a relatively trivial matter to add other materials into the silicone elastomer in its uncured state, and this has been exploited in a variety of ways, including improving the selectivity of the laser machining. A particular advantage of this approach is the ability to tune the absorption of the silicone to facilitate ablation of the top silicone layer without damaging other layers below (particularly metals).^[^ ^100^, ^317^ ^]^ Other amendments that have been added to silicones include carbon forms (Section 4.2.7), liquid metals (Section 4.2.5), and conducting polymers.^[^ ^173^ ^]^

Laser machining has also been used a number of times to pattern channels into a silicone layer, rather than cut all the way through the layer, to act as a microfluidic track, most commonly for lab‐on‐a‐chip applications.^[^ ^318^, ^319^ ^]^ Laser surface patterning of silicones has also been used for controlling wettability (to both “standard” liquids and liquid metals as discussed above),^[^ ^320^, ^321^, ^322^ ^]^ for cell adhesion and growth control,^[^ ^323^, ^324^ ^]^ and to selectively activate the surface for electroless deposition of metals in an additive manufacturing approach to bioelectronics.^[^ ^95^, ^325^, ^326^, ^327^ ^]^

Finally, through a pyrolytic process similar to the creation of LIG, it has been shown that the surface of a silicone material can be converted into conductive silicon carbide, which was used as both electrodes and interconnect tracks in implantable electrophysiological devices (Figure 7G).^[^ ^274^ ^]^

### 4.4 Microneedles

The noncontact nature of laser machining makes it an attractive option for several nonplanar applications. One popular use in biomedical settings is the creation of microneedles for a variety of biomedical applications. Lasers can be used in two different ways for microneedle fabrication: laser drilling of holes in a material to create a master mold from which polymer microneedles can be cast; and direct cutting and shaping of the material to form the microneedles. Laser fabrication of the master mold is by far the more common approach as it allows rapid creation of multiple copies of the desired device. However, direct shaping has proven popular as a low‐cost alternative to high resolution CNC machining and electrochemical etching for rigid materials. One emerging additional use of laser machining for microneedles was demonstrated by Abbasiasl et al. who used a UV femtosecond laser system to drill microchannels into pre‐formed microneedles to serve as a microfluidic delivery and sampling channel, Figure 7H.^[^ ^275^ ^]^ For further discussion on the role of laser machining in microneedle fabrication, the reader is directed to the many recent reviews on the topic.^[^ ^328^, ^329^, ^330^, ^331^, ^332^ ^]^

### 4.5 Stent Electrodes

An area of bioelectronics that is indirectly reliant on laser machining is the stent‐electrode, or “stentrode,” developed by the teams of Oxley and Opie (Figure 6M).^[^ ^96^ ^]^ These devices aim to facilitate minimally invasive neural interfacing by approaching neural tissue through the vascular system. Stentrode fabrication involves integrating metal recording electrodes and wires onto a commercially available cardiovascular stent. These stents themselves are laser‐machined from microtubes of a shape memory alloy, nitinol. While the stentrode, now a commercially available product, is based on laser‐machined commercially available nitinol stents, there has been considerable development in the laser‐machining of stents more generally, based on both laser cutting of preformed microtubes and on powder bed fusion of material to effectively 3D print stents.^[^ ^333^ ^]^ Beyond pure stent fabrication, there is interesting work leveraging LIPSS to alter the surface of the stent material to tailor its interaction with the surrounding tissue.^[^ ^159^ ^]^

Given the recent development of laser capabilities in academic bioelectronics groups globally, we expect to see further developments in this area. Leveraging the fabrication capabilities afforded by, in particular, ultrafast‐pulse lasers, we believe it is likely that stentrode‐like devices with tailored macro‐ and nanostructures will emerge, with seamlessly integrated electronics, created from both metal and polymer materials.

## 5 The Future: Challenges and Opportunities

Here, we have highlighted the use of laser micromachining to cut, ablate, and modify a range of materials for myriad bioelectronic applications. However, there remain several key challenges inhibiting the widespread adoption of laser processing.

Historically, one of the biggest challenges in laser machining, particularly for flexible electronics, has been the control of thermal damage to the soft polymeric materials. As discussed above, the use of ultrashort‐pulse lasers has greatly reduced these effects, allowing the processing of multiple layers with negligible thermal effects. However, these ultrashort‐pulse laser systems are still uncommon, particularly in academic contexts, owing to their cost and relatively recent development. While only a few publications have so far reported on their use in the fabrication of bioelectronic devices, the authors of this review are aware of a growing number of research groups across the globe who have invested in new systems for this purpose. In addition, the reduction in thermal effects has opened up the possibility of structuring hydrogel materials for a wide range of biomedical applications, as recently reviewed by Ma et al.^[^ ^334^ ^]^ As these systems start to become more widely available, it can be expected that laser machining will be applied widely across bioelectronics, and the wider flexible electronics fields, integrating hydrogels alongside more standard metallic and polymeric layers.

The proliferation of ultrashort‐pulse laser systems, particularly when coupled with appropriate optics, also presents an opportunity for the bioelectronics field in the form of the creation of embedded structures. For this, inspiration can be taken from the fields of photonics, where such lasers are used to directly write optical components such as waveguides and even microfluidic structures into solid optical materials like glass, silicon, or diamond.^[^ ^335^, ^336^, ^337^, ^338^, ^339^ ^]^ In addition to the short‐pulse lengths, this approach to laser machining is enabled by optics which produce highly confined focal spots with strong divergence away from the focal position, and so allow precise 3D patterning. This same process is expected to be possible with other materials relevant to bioelectronic applications, but to date only a very few works have shown this.^[^ ^340^, ^341^ ^]^ As ultrashort‐pulse systems become more available, we expect to see an increase in work looking at direct writing of structures embedded in polymer materials for both microfluidic and MEMS applications.

A challenge that has slowed the industrial adoption of laser micromachining, across all electronics sectors, is the scaling of laser processes from lab to commercialization. While laser micromachining is used for some commercial bioelectronics production, this is limited to low throughput, high value devices, for example the work of CorTec GmbH discussed in Section 4.1. For laser machining to become a viable alternative to photolithography on an industrial scale, further development of automation and high‐volume processing is required. The most promising avenue for industrial scale‐up is the use of roll‐to‐roll processing, compatible with both traditional deposition and printing processes. Development of this has been driven by the optoelectronics community, with commercial solutions now becoming available for wider flexible electronics applications,^[^ ^342^, ^343^, ^344^, ^345^ ^]^ promising high processing speeds at micrometer‐level positioning accuracy. Another development towards the scalability of laser processing is the introduction of multilaser systems. This may be the splitting of a single laser beam into multiple spots for parallel processing,^[^ ^346^, ^347^ ^]^ which has been shown to be capable of processing areas of up to 1 m^2^ min^−1^ in a roll‐to‐roll configuration,^[^ ^348^ ^]^ or the introduction of more than one laser, with different wavelengths and/or pulse lengths into a single system to enable processing of different materials simultaneously.^[^ ^349^ ^]^ To date, both roll‐to‐roll and multibeam systems remain uncommon due to both their cost and their size. Commercializing laser micromachining for bioelectronics will depend on the availability of these technologies at scale, which in turn will require national‐level support for bioelectronics manufacturing.

The development of laser machining can also benefit from the recent explosion in machine learning.^[^ ^350^ ^]^ Unlike other fabrication approaches, laser processing conditions can be iterated on rapidly during process optimization. However, laser machining also has a wider parameter space than other approaches. This combination can lead to a plurality of data that is hard to evaluate using standard approaches, but which is ideal for machine learning. By properly characterizing the parameter space, and combining this with advanced imaging techniques, it is likely that bespoke laser manufacturing processes can be developed for myriad applications. In addition, these machine learning techniques can allow real‐time monitoring of the machining process based on the appearance of the generated plasma, providing a way of ensuring optimal results even when materials defects may be present. There have been a number of recent demonstrations of this approach for optimizing and monitoring of laser machining,^[^ ^351^, ^352^, ^353^, ^354^ ^]^ though these are yet to be translated to general use. To make this approach truly applicable, the field must first mature to the point where a broad range of materials can be used and real‐time error correction is possible,^[^ ^355^ ^]^ and second, such technology must be integrated into commercially available systems, suitable for nonspecialists.

## 6 Conclusions and Outlook

We have seen here that laser processing, while still a relatively immature technique, is starting to gain traction in bioelectronics fabrication. While LIG remains the most widespread use of laser processing in this community, the last five years have seen the proliferation of other applications of lasers as shorter wavelength and pulse length lasers become more accessible. This has seen the development of conductive polymer processing, both to ablatively pattern the material and to induce fundamental material characteristic changes to control electromechanical properties.

As laser technology has evolved from early excimer lasers, with low resolution and primitive patterning technology, through the proliferation of low‐cost CO_2_ lasers ideal for cutting individual foils, and on to current short and ultrashort pulse systems with high‐speed, high‐precision optics, we have seen the development of a range of laser machined bioelectronics.

The development of bioelectronics based on laser machining has shadowed the development of the laser systems themselves. Early adoption was limited to cutting individual layers at relatively low resolution due to low selectivity and poor focal control. As the optics used with lasers improved, the resolution of devices increased, leading to the creation of high channel counts for neural interfaces, though still mostly based on relatively thick materials best suited to cutting with excimer or CO_2_ lasers. The introduction of fiber and semiconductor lasers, with a broad wavelength range across the visible spectrum, and the development of shorter pulse lasers enabled a proliferation of laser‐based manufacturing in bioelectronics, with material selectivity allowing multilayer processing, particularly of thin‐film metals on polymer substrates, and even selective material characteristics modification. Now, with the next generation of laser systems, featuring ultrashort pulse lengths and advanced optics systems, near any material can be patterned. Conducting layers can be patterning into arbitrary electrode and interconnect shapes, in 2D and 3D devices, on diverse and nonplanar substrates, limited only by the resolution of the laser beam, and insulating materials, can be cut, milled, welded, and surface patterned to create myriad device types. While innovation with this new generation of laser systems is currently limited, it is expected to rise rapidly as they become more available.

From this review, we hope that the reader has gained a strong understanding of the different applications of laser processing to date, as well as a feel for the key processing parameters for different materials and device structures. Throughout, we have tried to highlight the enabling factors that make laser processing both possible and advantageous for different applications, with the application overviews serving as a potential reference point when considering the use of laser processing in one's own work.

The most recent move has been towards ultra‐short‐pulse systems capable of overcoming the remaining materials limitations, and the integration of advanced optical components pushing laser patterning towards the resolution limit set by optical diffraction. As ultrashort‐pulse systems become more common, and technological improvements continue we are confident we will see a step change in the development of new biomedical technologies developed through laser micromachining. As these systems are further refined, throughput increased, and commercialization of production enabled, we expect to see laser processing become common throughout the bioelectronics realm. The future of lasers in bioelectronics is bright indeed.

## Conflict of Interest

The authors declare no conflict of interest.
